# Redo Total Aortic Arch Replacement in Patients with Aortic Dissection After Open-Heart Surgery and Long-Term Follow-Up Results

**DOI:** 10.21470/1678-9741-2022-0022

**Published:** 2023

**Authors:** Feng Gao, Yipeng Ge, Yongliang Zhong, Xijing Zhuang, Junming Zhu

**Affiliations:** 1 Department of Cardiovascular Surgery, Beijing Anzhen Hospital, Capital Medical University, Beijing Aortic Disease Center, Beijing Institute of Heart, Lung and Blood Vessel Diseases, Beijing Engineering Research Center of Vascular Prostheses, Beijing, Chaoyang District, People’s Republic of China; 2 Department of Cardiology Surgery, Dalian Municipal Central Hospital, Dalian, Liaoning Province, People’s Republic of China

**Keywords:** Cardiac Output, Low, Risk Factors, Aneurysm, Dissecting, Coma, Extracorporeal Circulation, Perioperative Period.

## Abstract

**Introduction:**

The objectives of this study were to investigate the main treatment strategies and long-term follow-up results of aortic dissection surgery after open-heart surgery (ADSOHS) and to analyze the risk factors that cause ADSOHS.

**Methods:**

One hundred thirty-seven patients with ADSOHS hospitalized in our hospital from January 2009 to December 2018 were selected as the research object. Long-term follow-up results, complications, mortality, and changes of cardiac function before and after operation were used to explore the value of Sun’s operation.

**Results:**

The length of stay in intensive care unit of these 137 patients ranged from 9.5 to 623.75 hours (average of 76.41±97.29 hours), auxiliary ventilation time ranged from 6.0 to 259.83 hours (average of 46.16±55.59 hours), and hospital stay ranged from six to 85 days (average of 25.06±13.04 days). There were seven cases of postoperative low cardiac output, 18 cases of coma and stroke, and six cases of transient neurological dysfunction. A total of 33 patients died; 19 patients died during the perioperative period, 18 died during Sun’s operation and one died during other operation; and 14 patients died during follow-up (January 2021), 12 cases of Sun’s operation and two cases of other operations.

**Conclusion:**

ADSOHS treatment strategy is of high application value, and the risk of neurological complications and mortality is low. The main risk factors are postoperative low cardiac output, coma, stroke, and transient neurological dysfunction. The extracorporeal circulation time is relatively long. Short- and long-term follow-up effects are good, and it is worthy of clinical promotion.

## INTRODUCTION

Aortic dissection surgery after open-heart surgery (ADSOHS) is rare in clinical practice, but the history and etiology of this disease is complex. How to avoid the occurrence and formulating surgical strategies are some of the difficulties in cardiac surgery^[^1^]^. The incidence of aortic dissection is 6/100,000, and it is more common in men than women^[^2^]^. Most patients with aortic coarctation die within a few hours to days of onset, and the hourly mortality rate within the first 24 hours is 1-2%, depending on the location, extent, and degree of the disease^[^3^]^. Jassar et al.^[^4^]^ studied 120 patients with root replacement with a history of previous cardiac surgery (PCS) and found that age, diabetes, coronary artery bypass grafting, and endocarditis were risk factors for increased hospital mortality. There are relatively few studies on ADSOHS in China. Professor Wang Chunsheng’s team reported 28 cases of acute aortic dissection combined with a history of cardiac surgery over a 10-year period. Mortality within 30 days after surgery reached 21.4%, low cardiac output syndrome, neurological system damage, and multiorgan failure being the main causes of death^[^4^,^5^]^. In the current clinical research reported on the treatment of ADSOHS, surgery still has a high risk of complications and mortality. The safety and effectiveness of surgical treatment need to be further explored^[^6^]^. This study aimed to explore the conventional surgical strategies and long-term follow-up results of ADSOHS treatment to provide clinical references.

## METHODS

### General Information

We performed a retrospective study of data of 137 patients who were hospitalized in the Department of Cardiovascular Surgery of Beijing Anzhen hospital between January 2009 to December 2018 and who underwent Sun’s surgery.

Inclusion criteria were: patients diagnosed with aortic dissection^[^6^]^; patients undergoing midline thoracotomy due to aortic dissection; previous history of open-heart surgery; patient’s dissection involving the aortic arch and descending aorta; and patients without contraindications to surgery and whose families signed informed consent forms.

Exclusion criteria were: emergency patients non-hospitalized for any reason or death before hospitalization; patients with significant medically induced aortic coarctation, such as percutaneous coronary intervention, cannulation, surgery, etc; patients with intestinal hemorrhage and necrosis caused by the superior mesenteric artery of the abdomen; patients suffering from insufficient blood supply to the myocardial coronary artery and large-area myocardial infarction; inadequate blood supply to the brain leading to coma and other neurological problems; insufficient coagulation or cognitive-communication impairment; patients who failed to follow-up or missing data during follow-up; and simple involvement of the ascending aorta.

### Quality Control Methods

Relevant research data were collected in a face-to-face and follow-up manner: the collection of clinical data was performed by one person, independently, and the data was reviewed and quality controlled by a dedicated person; the image reading and data measurement phases of imaging-related clinical data were performed by two or more medical professionals, and a third person verified it to avoid mistakes; two people were present during the follow-up to ensure that the follow-up results were authentic and credible; the supervisory team and the Aorta Centre research staff supervised the project.

### Gathering Information

The study collected complete data for the next step, including inspection data (blood type, admission international normalized ratio, admission white blood cell count, admission creatinine [Cr], pre-discharge Cr, preoperative ascending aortic diameter, preoperative left ventricular end-diastolic transverse diameter, preoperative left ventricular ejection fraction [LVEF], preoperative aortic valve regurgitation, preoperative aortic valve stenosis, postoperative LVEF, and postoperative left ventricular end-diastolic transverse diameter), surgery-related data (preoperative circulatory stability, preoperative tracheal intubation, preoperative chest pain, preoperative pericardial tamponade, dissection rupture, preoperative hemiplegia, preoperative loss of consciousness, preoperative paraplegia, preoperative abdominal ischemia, preoperative lower limb ischemia, conservative treatment and causes, surgical urgency, surgical approach, concomitant other operations, rupture orifice position, number of breaches, arch involvement, brachiocephalic vascular involvement, arterial cannulation position, venous cannulation position, perfusion fluid, extracorporeal circulation time, aortic occlusion time, circulatory arrest time or selective cerebral perfusion time, and minimum body temperature), and postoperative-related data (surgical blood usage [red blood cells, platelets, plasma], postoperative blood loss, second thoracotomy haemostasis, duration of intensive care unit [ICU] stay, postoperative duration of assisted ventilation, postoperative blood purification, postoperative low cardiac output, postoperative extracorporeal membrane oxygenation, postoperative coma and stroke, transient neurological dysfunction, paraplegia, in-hospital death, total cost, follow-up time, reoperation during follow-up, and death during follow-up).

### Sun’s Surgical Method

After anesthesia, a sterile drape was usually placed to adequately expose the surgical area. A small incision was made approximately 1 cm below the midline of the right clavicle, the right axillary artery was isolated and revealed, and the artificial vessel was anastomosed to the end of the axillary artery. The thoracic cavity was incised in the middle of the sternum to free the three main branches of the arterial arch. The drainage tube was inserted into the right atrium to complete the circulatory drainage. Left ventricular drainage was completed by intubation through right superior pulmonary vein. The patient’s body became hypothermic. When the temperature reached 32ºC, the ascending aorta was clamped, and the ascending aortic root was analyzed and treated by passing through the coronary artery starting point to inject myocardial protective fluid. When the nasopharyngeal temperature drops to 18-20ºC and the anal temperature is 25ºC, low flow at 5-10 ml/(kg.min) [7.85±1.76 ml/(kg.min)] selective cerebral perfusion occurs in the innominate, left common carotid, and left subclavian and right axillary arteries, stopping the circulation (34.77±10.25 min). Disconnect the ascending aorta, disconnect the aortic arch from the distal end of the left subclavian artery, and pay attention to protecting the vagus nerve around the arch. A suitable type of stent vessel (truss) was passed distal to the aortic arch to be implanted into the true lumen of the descending aorta. A trunk stent of comparable diameter to the stent vessel was selected and “sandwich” sutured to the distal end of the quadrant prosthesis at its proximal end and the proximal end of the descending aorta. The perfusion branch of the artificial blood vessel was inserted into the other end of the arterial pumping tube and evacuated of gas. The corresponding branch of the artificial vessel was anastomosed to the end of the left common carotid artery. After exhaustion, cerebral perfusion was first restored, followed by proximal anastomosis, lung expansion, and opening of the ascending aorta. Then the left subclavian artery and the innominate artery were withdrawn in sequence, being cut off, and the perfusion branches were ligated, followed by opening of the obstruction and closing of the thoracic cavity.

### Observation Indicators

- Describe and analyze the PCS of patients requiring ADSOHS and the reasons for it;
- Analyze and summarize the treatment strategy, efficacy, and long-term follow-up results of ADSOHS in our center;
- Explore the risk factors for ADSOHS formation and surgical risk factors;
- Patient follow-up: all registered patients were followed up by telephone at six months postoperatively. For patients who could not be followed up by telephone, on-site visits were conducted according to the patient’s admission registration address, and the patient’s previous information and follow-up information were recorded.

### Statistics

IBM Corp. Released 2017, IBM SPSS Statistics for Windows, version 25.0, Armonk, NY: IBM Corp. was used for data processing with a test level of ɑ=0.05. Statistical description, independent samples *t*-test, one-way analysis of variance, chi-squared test, and Kaplan-Meier survival analysis were performed for statistical analysis.

## RESULTS

### Normal Information

Patients’ data: 112 males and 25 females, mean age of 44.51±11.43 years old (range 20-69 years), average height of 157.52±39.67 cm (range 147-200 cm), average weight of 68.53±17.43 kg (range 39-98 kg), and basic complications - 96 cases of hypertension, nine cases of Marfan disease, and 10 cases of acute myocardial infarction within three weeks (Table 1).

**Table 1:** Patient information.

Group		Numerical value
Gender	Male	112
	Female	25
Average age (years)		44.51±11.43
Average height (cm)		157.52±39.67
Average weight (kg)		68.53±17.43
Basic complications	Hypertension	96
	Marfan disease	9
	Acute myocardial infarction within 3 weeks	10

### Intraoperative Condition of Sun’s Surgery

Treatment for type A aortic dissection mainly include medication to control blood pressure, analgesic sedation, supportive treatment such as strict bed rest, Sun’s surgery, and debranching hybrid surgery. Patients should have relevant investigations completed as soon as possible after admission, such as echocardiography, electrocardiogram, whole aortic computed tomography angiography, liver and kidney function, blood and urine routine, coagulation tetralogy, and infectious diseases. Among them, those with obvious tear-like pain and a history of hypertension should be highly suspicious of the possibility of aortic coarctation. At this time, appropriate anti-hypertensive medication should be given to prevent aortic dissection due to hypertension and lost opportunity for further surgery. Meanwhile, morphine can be given for intramuscular analgesia, and narcotics can be used for sedation. Those with irritability, haemodynamic instability, and poor analgesic sedation with medication should be urgently intubated or tracheotomized and prepared for emergency surgery. The greatest advantage of Sun’s procedure was that it allows creative transcription of the left distal common carotid artery and the left subclavian artery, changing the disadvantages of the traditional elephant trunk procedure.

**Fig. 1:**
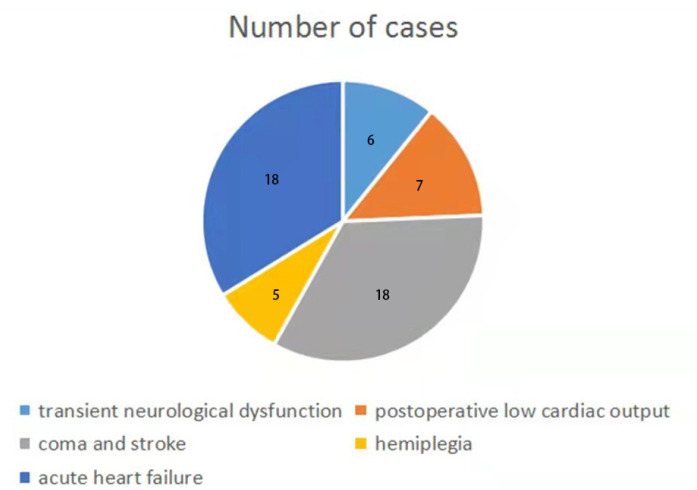
Postoperative negative outcomes.

Emergency surgery was performed in 31 cases (22.87%), elective surgery in 106 cases (77.13%), and there was no reoperation. Rupture site: sinus and aortic arch in 21 cases (15.43%), innominate artery and opening in five cases (3.72%), and left subclavian artery in five cases (3.72%). Postoperative negative outcomes are shown in Figure 1. Cardiopulmonary bypass time ranged from 107 to 450 minutes (average time 193.23±57.23 minutes), active pulse occlusion time ranged from 37 to 203 minutes (average time 95.28±31.55 minutes), and cerebral perfusion time ranged from 0 to 60 minutes, with two means of 25.38±10.51 minutes.

### Perioperative Situation

ICU stay time ranged from 9.5 to 623.75 hours (average time 76.41±997.29 hours), ventilation assistance time ranged from six to 259.83 hours (average time 46.16±55.59 hours), and hospital stay ranged from six to 85 days (mean of 25.06±13.04 days). There were postoperative low cardiac output in seven cases (5.32%), 18 cases of coma and stroke (13.30%), and transient neurological deficits in six cases (4.26%) (Table 2).

**Table 2:** Perioperative situation.

Group		Numerical value
ICU stay average time (hours)		76.41±97.29
Ventilation assistance average time (hours)		46.16±55.59
Hospital stay average time (days)		25.06±13.07
Complications	Postoperative low cardiac output	7
	Coma and stroke	18
	Transient neurological dysfunction	6

ICU=intensive care unit

Perioperative death: 19 cases; 18 cases during Sun’s surgery and one case during other surgery. The specific causes of death are as follows: three patients died of postoperative low cardiac output and heart failure; two patients died of intraoperative aortic rupture and low cardiac output; seven patients died of postoperative renal failure, liver failure, and multiple cerebral infarctions; three patients died of postoperative bleeding, two had openings to stop bleeding; and four patients died of postoperative cerebral infarction complicated with intracerebral hemorrhage (Table 3).

**Table 3:** Perioperative and follow-up death.

Group		Numerical value
Perioperative death	Postoperative low cardiac output, heart failure	3
	Intraoperative aortic rupture, low cardiac output	2
	Multiple organ failure, cardiac arrest, and death	6
	Postoperative brain infarction accompanied by cerebral hemorrhage	4
Follow-up death	Severe mitral regurgitation	3
	Severe tricuspid regurgitation	1
	Severe pulmonary hypertension	2
	Left ventricular hypertrophy	2
	Multiple organ failure, cardiac arrest, and death	1
	Postoperative low cardiac output, heart failure	4
	Postoperative brain infarction accompanied by cerebral hemorrhage	1

### Follow-Up Results

**Fig. 2:**
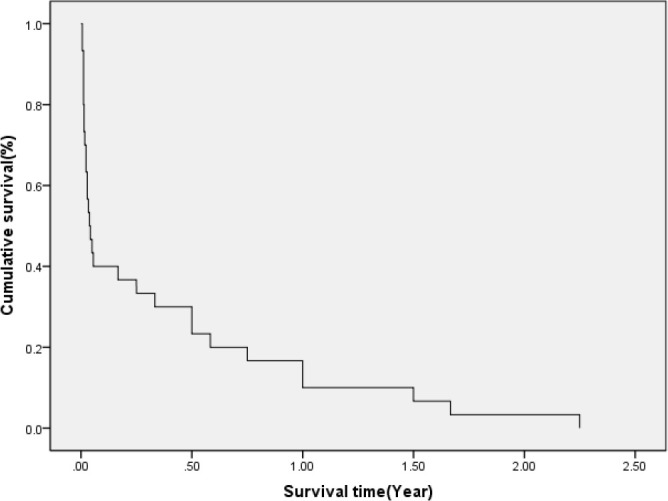
Survival rates of the operated patients.

Follow-up death (from January 2021 to August 2021): 14 patients died during the follow-up period; 12 cases of Sun’s surgery and two cases of other surgeries. The specific causes of death were as follows: four deaths due to postoperative low cardiac output and heart failure; one death due to postoperative renal failure, liver failure, and multiple cerebral infarction; one death due to cerebral infarction combined with cerebral haemorrhage; three deaths due to severe mitral regurgitation; one death due to severe tricuspid regurgitation; two deaths due to severe pulmonary hypertension; and two deaths due to left ventricular hypertrophy (Table 3). The survival rates of the operated patients are shown in Figure 2.

## DISCUSSION

Aortic dissection is a disease that seriously endangers human health, and it is also one of the most complicated diseases in the surgical system^[^7^,^8^]^. The disease is like a time bomb buried deep into the human cardiovascular system, which may burst at any time and cause death. Type A aortic dissection in the Stanford classification has a mortality rate of > 50% within 48 hours and up to 90% within two weeks. Once the disease occurs, emergency surgery is required; many patients die because of sudden onset, too late to see a doctor, and rupture of the dissection causing sudden pericardial tamponade, circulatory failure, and ischaemia in several vital organs of the human body^[^9^]^. Timely surgery can reduce the in-hospital mortality rate to 27%, while the mortality rate of conservative medical treatment alone is close to 60%. Intervention of this disease through surgery has become a worldwide consensus and the most important treatment at present^[^10^]^.

In this study, Sun’s postoperative mortality rate after surgical treatment was 2.66%, significantly lower than 20% in foreign literature, and only one death occurred during follow-up. The surgical treatment of aortic coarctation by Sun’s procedure was confirmed to reduce perioperative mortality. The reason for this is that Sun’s procedure has the greatest advantage of creatively transcribing the distal left common carotid artery and the left subclavian artery, which changes the disadvantages of the traditional elephant trunk surgery. To a large extent, it reduces neurological complications and extracorporeal circulation time^[^11^]^. The use of free artificial blood vessels in the descending aorta during conventional aortic dissection elephant trunk surgery may affect retrograde perfusion. The elephant trunk surgery is mainly to expand the pressure to reduce the true cavity of the aorta, so that the blood flow in the false cavity is slowed down and thrombosis is formed^[^12^,^13^]^.

In the present study, this method has certain disadvantages, with a tendency to cause paraplegia and organ embolism after surgery. And other symptoms. Sun’s procedure allows for resection of the fissure while minimizing dilatation and recurrence of diseased blood vessels^[^14^]^. The analysis of complications shows that acute renal failure and neurological disorders are the most common complications. The occurrence of acute renal failure following type A aortic coarctation is mainly related to the following factors: first, it is caused by prolonged cardiopulmonary extracorporeal circulation. Then, massive production of inflammatory reaction and inflammatory mediators, various inflammatory mediators, and sympathetic excitation stimulate intraoperative blood redistribution, peripheral vascular - especially renal vascular - constriction, reduce renal blood flow, reduce renal perfusion pressure, and stimulate the onset of renal failure; if this state persists and cannot be corrected, it will develop into renal acute renal failure^[^15^]^. In order to reduce the incidence of acute renal failure from this reason, we should continuously improve the surgical technique, shorten the extracorporeal circulation time, and reduce the production of inflammatory mediators. Therefore, in clinical work, whether it is preoperative, intraoperative, or after surgery, the patient’s vital signs should be closely observed to maintain hemodynamic stability, especially to prevent renal hypoperfusion and acute renal failure caused by low blood pressure^[^16^]^.

### Limitations

Because the sample of this study is the patients who underwent aortic dissection surgery for the second time, the number of patients in this clinical study is not sufficient due to the severity of the disease. Some patients have a short follow-up time, and there may be some survivor bias.

## CONCLUSION

In recent years, the postoperative mortality rate of aortic coarctation has been significantly lower than before, but postoperative complications still affect the efficacy of surgery and lead to patient’s death. Therefore, we must conduct a comprehensive analysis of the possible postoperative complications in our clinical work and manage them correctly in order to minimize their occurrence.
